# *Lactobacilli*-Mediated Regulation of the Microbial–Immune Axis: A Review of Key Mechanisms, Influencing Factors, and Application Prospects

**DOI:** 10.3390/foods14101763

**Published:** 2025-05-16

**Authors:** Hong-Fei Ji, Mei Li, Xiao Han, Yu-Ting Fan, Jia-Jing Yang, Yan Long, Juan Yu, Hai-Yu Ji

**Affiliations:** Yantai Key Laboratory of Characteristic Agricultural Bioresource Conservation & Germplasm Innovative Utilization, School of Life Sciences, Yantai University, Yantai 264005, China; hongfei10052024@163.com (H.-F.J.); mayli113@ytu.edu.cn (M.L.); hanxiao7721@163.com (X.H.); fyting7766@163.com (Y.-T.F.); yangfan2812@163.com (J.-J.Y.); 18282780162@163.com (Y.L.); yj0805@ytu.edu.cn (J.Y.)

**Keywords:** *Lactobacilli*, immunomodulatory mechanisms, application strategies

## Abstract

*Lactobacilli*, as the main member of food microorganisms, is an important component of the intestinal microbial community and plays crucial roles in regulating the immune capacity of the body. This review provides a comprehensive exploration of the key components of *Lactobacilli*-mediated immune regulation effects, including the immunogenic components (peptidoglycan and lipoteichoic acid) and metabolites (short-chain fatty acids, bacteriocins, and exopolysaccharides), which can interact with host immune cell receptors to initiate complex immune signaling pathways. In addition, the immunomodulatory activity can be influenced by multiple factors including species differences, host-related factors (age, physiological conditions, and gut microbiota), and environmental factors (nutrient substrates, temperatures, etc.), and the application strategies including precision probiotic development, gene-editing driven engineering, and nanocarrier systems have also been proposed to enhance the immunomodulatory potential. Finally, this review provides the theoretical basis for microbial intervention in immune-related diseases and offers prospects for applications in the food and pharmaceutical industries.

## 1. Introduction

In recent years, natural bioactive substances, owing to their remarkable immunomodulatory properties, have become a contemporary research hotspot due to their applications in the development of functional foods [1,2]. As reported, various polysaccharides and proteins have exhibited strong immunomodulatory effects [3,4]. At the cellular level, these compounds can enhance macrophage phagocytic capacities, facilitate dendritic cell-mediated antigen presentation to T lymphocytes [5], and regulate cytokine secretion including interleukins, interferons, etc. [6]. However, as dietary components, these macromolecular active substances are unable to directly enter the bloodstream and can only interact with gut microorganisms and a limited number of immune cells present in the intestinal environment. Therefore, the underlying mechanisms of indirect immune regulation through remodeling intestinal microecology should not be overlooked [7,8].

*Lactobacilli*, principal members of the *Lactobacillaceae* family, are Gram-positive facultative anaerobic bacteria and colonize mucosal surfaces within the gastrointestinal, oral, and genitourinary tracts of host organisms [9], which can metabolize carbohydrates via the glycolytic pathway and produce organic acids, thereby impeding the proliferation of pathogenic microbes [10]. It is important to highlight that the genus of *Lactobacilli* underwent a major revision in 2020, which was divided into over 20 new genera, and relevant information can be indexed in the List of Prokaryote Names with Standing in Nomenclature (LPSN, available at https://lpsn.dsmz.de (accessed on 10 May 2025)). Up to now, there are still incorrect or outdated strain descriptions in many database research results. Therefore, the *Lactobacilli* in this paper encompass both the *Lactobacillus* genus and the new genera derived from it.

*Lactobacilli* have exhibited multiple functional roles encompassing immunomodulation, metabolic disease intervention, and neuroimmune axis regulation [11,12,13,14]. As reported, *L. acidophilus* and *L. rhamnosus* can modulate gut microbiota compositions, reinforce mucosal barrier integrity, and regulate systemic immune responses [15,16]. In addition, the *Lactobacilli* and natural bioactive compounds have significant synergistic effects in immune regulation. Exogenous polysaccharides (including dietary fiber), acting as prebiotics, have been shown to selectively promote *Lactobacilli* proliferation and intestinal colonization, thereby augmenting the immunomodulatory efficacy [17]. In addition, the *Lactobacilli* metabolites can also enhance the bioactivity of natural compounds, creating a reciprocal amplification loop [18]. The immunomodulatory properties of *Lactobacilli* are characterized by multitargeted actions, low cytotoxicity, and microecological synergy [19], which can be delineated into two primary dimensions: direct bacteria–host interactions and indirect metabolite-mediated regulation [20,21]. Advancements in multi-omics technologies have gradually revealed the molecular basis of *Lactobacilli*-mediated immunoregulation, enabling unprecedented insights into microbial–host interactions [22,23].

This review summarizes the immune regulatory mechanisms of bacterial components of *Lactobacilli* (such as peptidoglycan and lipoteichoic acid) and their metabolites (such as short-chain fatty acids and bacteriocins), analyzes the effects of strain-specific differences, host-related factors, and environmental influences on immunomodulatory outcomes, outlines the application strategies for immune intervention such as the precise development of probiotics, synthetic biology-based gene modifications, and the construction of metabolite delivery systems. This paper can provide the theoretical basis for exploring the mechanisms of *Lactobacilli*-mediated immunomodulation of functional foods, and further facilitate relevant research, developments, and applications of *Lactobacilli*-related products in the food and pharmaceutical industries.

## 2. Immunoregulatory Mechanisms of Lactobacilli

**Figure 1:**
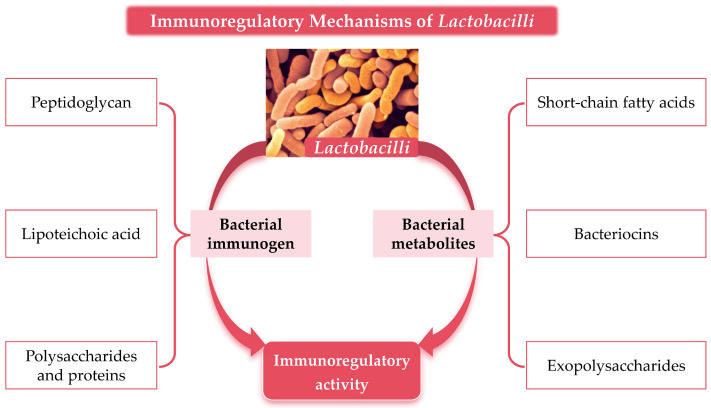
Material basis of *Lactobacilli*-mediated immunomodulatory activity.

As an important group of probiotics, *Lactobacilli* mediate the immunomodulatory mechanisms primarily through two major pathways: their own components (immunogenic substances) and metabolites, as shown in Figure 1.

### 2.1. Immunoregulatory Mechanisms of Lactobacilli Immunogen

*Lactobacilli* are composed of the cell wall, cell membrane, cytoplasm, and other subcellular components. Notably, the cell walls play pivotal roles in immunoregulatory processes, which are predominantly constituted by peptidoglycan, lipoteichoic acid, polysaccharides, and other bioactive molecules [24]. These components can mediate interactions with pattern recognition receptors (PRRs) on the host immune cell surface, thereby initiating immune signaling and transduction pathways that orchestrate both innate and adaptive immune responses [25,26].

#### 2.1.1. Peptidoglycan

Peptidoglycan (PG), a major component of *Lactobacilli* cell walls, consists of a glycan backbone interconnected by tetrapeptide side chains and peptide bridges, forming a highly conserved macromolecular lattice [27]. The immunomodulatory properties of PG are closely related to the integrity of the macromolecular structure, the composition of glycan chains, and the amino acid sequence of tetrapeptide side chains [28].

Host immune cells can recognize *Lactobacilli* PG through NOD-like receptors, with NOD1 and NOD2 serving as the primary sensors. NOD1 selectively detects PG fragments containing γ-D-glutamyl-m-diaminoheptanedioic acid, whereas NOD2 is specialized for recognizing muramyl dipeptide moieties [29,30,31]. Ligand binding to NOD1 or NOD2 initiates a signaling cascade culminating in the activation of the serine/threonine kinase [32], which triggers the phosphorylation and subsequent degradation of the inhibitory protein, releasing nuclear factor-κB (NF-κB) from cytosolic sequestration. Translocated NF-κB then moves to the nucleus and binds to promoter regions of target genes, inducing the transcription of tumor necrosis factor-α and interleukin-6, thereby augmenting the immune responses [33,34]. In addition, *Lactobacilli* PG also exerts precise regulation over immune cell activation, proliferation, and differentiation through mitogen-activated protein kinase (MAPK) signaling pathways [35,36].

Structural and functional disparities in PG have been observed among different *Lactobacilli* strains. PG derived from human intestinal isolates often exhibits tetrapeptide side-chain configurations that more effectively engage immune cell receptors, conferring superior immunostimulatory capacities compared to strains from non-host environments [37]. Additionally, variations in PG preparation methodologies significantly impact the structural integrity, thereby influencing receptor binding affinity and subsequent signaling efficacy [38].

#### 2.1.2. Lipoteichoic Acid

Lipoteichoic acid (LTA), a distinctive constituent of the Gram-positive bacterial cell wall, is composed of phosphoglycerol residues covalently linked via phosphodiester bonds. LTA is a critical interface for host–microbe interactions with one terminus anchoring to the cytoplasmic membrane through lipid moieties, while the opposing end extends outward to the cell wall surface [39]. Structural and compositional variations in LTA among *Lactobacilli* strains contribute to diverse immunomodulatory activities, underscoring the effects of strain-specific nature [40].

The immune recognition of LTA is mediated by Toll-like receptor (TLR) 2 on host immune cells, which can form heterodimeric complexes with TLR1 or TLR6, and then recruit myeloid differentiation primary response 88 (MyD88) [41]. MyD88 can initiate downstream signaling cascades and activate NF-κB and MAPK pathways, which leads to the transcriptional induction of relevant cytokines and chemokines, thereby orchestrating regulatory immune responses [42]. LTA-stimulated immune cells often exhibit anti-inflammatory effects, such as promoting the secretion of interleukin-10, which can mitigate the excessive inflammation of immune responses [43]. Experimental evidence from mice models of inflammatory bowel disease demonstrates that *Lactobacilli* LTA treatment mitigates intestinal inflammation by upregulating interleukin-10 expression and downregulating pro-inflammatory cytokines such as tumor necrosis factor-α and interleukin-1β [44].

The immunomodulatory effects of LTA are further modulated by dosage and contextual factors. Low concentrations of LTA primarily induce anti-inflammatory effects, whereas higher doses may elicit pro-inflammatory responses under specific conditions [45]. Additionally, LTA can interact synergistically with interferon-γ and enhance the pathogen resistance of hosts while mitigating the uncontrolled inflammation causing tissue damage [46]. In addition, LTA can also regulate the maturation and functionality of dendritic cells [47] and enhance the capacities of antigen uptake, processing, presentation, and cytokine secretion, thereby potently enhancing T-cell proliferation and polarization toward specific subsets [48,49].

#### 2.1.3. Additional Immunomodulatory Components

Other constituents of *Lactobacilli* cell walls including polysaccharides and surface proteins can also significantly contribute to the immunomodulatory processes. As reported, *Lactobacilli* polysaccharides exhibit diverse immunomodulatory properties including enhancing macrophage phagocytic activity, natural killer cell cytotoxicity against pathogens, and stimulating the secretion of pro-inflammatory cytokines [50]. Specific *Lactobacilli* polysaccharides can interact with mannose receptors on macrophage surfaces, initiating intracellular signaling cascades involving MAPK and NF-κB pathways and triggering the upregulation of tumor necrosis factor-α and interleukin-1β [51]. In vitro experiments showed that *Lactobacilli* polysaccharides could significantly enhance the phagocytic capacities of macrophages with higher mRNA expression levels of tumor necrosis factor-α and interleukin-1β, indicating the roles in initiating innate immune responses [52,53].

Similarly, *Lactobacilli* surface proteins serve as critical immunomodulatory ligands, mediating interactions with host immune cell receptors. Adhesion proteins on the bacterial surface can bind to complementary receptors on intestinal epithelial cells or immune cells, thereby modulating intercellular communication and influencing immune cell function [54]. Studies have shown that certain *Lactobacilli* adhesion proteins can enhance the expressions of tight junction proteins in intestinal epithelial cells, reinforcing mucosal barrier integrity and regulating the activity of lamina propria lymphocytes, which can strengthen intestinal immune homeostasis [55,56].

Therefore, a variety of *Lactobacilli* components present immunogenic properties, which can activate the immune cells through multiple pathways. However, due to different strains, the structure and functions of immunogenic components showed significant differences. Future studies can employ advanced techniques such as high-resolution nuclear magnetic resonance and X-ray crystal diffraction to further analyze the molecular structure differences of active components such as peptidoglycan and lipoteichoic acid in different *Lactobacilli* strains. In addition, gene-editing and site-specific mutation can be used to change the structure of active components and explore the effects of structural changes on the physiological activities and immune regulatory functions of *Lactobacilli*. Through systematic and in-depth studies, the structural–activity relationship of *Lactobacilli* active components can be deeply understood at molecular levels, which will provide a solid theoretical basis for the development and applications of *Lactobacilli* and the components in the functional dietary adjuvants field.

### 2.2. Immunomodulatory Mechanisms of Lactobacilli Metabolites

*Lactobacilli* can produce various metabolites during growth and metabolism, including short-chain fatty acids, bacteriocins, exopolysaccharides, and others [57], which play crucial roles in regulating the intestinal microecological balance and influence the host’s immune system [58,59].

#### 2.2.1. Immunomodulatory Effects of Short-Chain Fatty Acids

Short-chain fatty acids, primarily acetic, propionic, and butyric acids, represent major metabolites generated during *Lactobacilli*-mediated carbohydrate fermentation [60,61], which has exhibited distinct immunomodulatory roles in hosts. The short-chain fatty acid-mediated immunomodulatory functions are exerted through two principal mechanisms: binding to G protein-coupled receptors and the modulation of histone deacetylase activity [62,63]. Intestinal epithelial cells and immune cells express various short-chain fatty acid-specific G protein-coupled receptors, including GPR41, GPR43, etc. [64]. The engagement with GPR41 triggers the activation of the phospholipase C-protein kinase C signaling axis, modulating intracellular calcium levels and influencing immune cell effector functions. While GPR43 activation dampens inflammatory responses by attenuating NF-κB signaling, thereby reducing pro-inflammatory cytokine production [65]. Clinical trials have demonstrated that supplementation with probiotic formulations rich in short-chain fatty acids ameliorates intestinal inflammation, which can decrease fecal pro-inflammatory cytokine levels [66].

Distinct short-chain fatty acid species exhibit specialized immunomodulatory effects [67,68]. Acetic acid primarily influences immune function indirectly by modulating the compositions and metabolic activity of intestinal microbiota [69]. Propionic acid inhibits hepatic cholesterol biosynthesis while regulating intestinal epithelial cell metabolism and functions, thereby influencing immune homeostasis [70,71]. Butyric acid assumes a central role in maintaining intestinal integrity and immune cell regulation, which can promote intestinal epithelial cell proliferation and differentiation, strengthens the mucosal barrier against pathogen invasion, and suppresses inflammation by fostering immune tolerance [72,73].

In addition, short-chain fatty acids also profoundly regulate the differentiation and functional polarization including enhancing the generation of regulatory T cells while inhibiting the differentiation of pro-inflammatory T helper 17 (Th17) cells, thereby preserving immune homeostasis [74]. In vitro studies have shown that butyric acid supplementation can increase regulatory T cell proportions and concomitantly reduce Th17 cell polarization [75], which is mediated mainly by the short-chain fatty acid-induced reprogramming of T cell metabolism. Butyric acid can promote fatty acid oxidative metabolism in Tregs, sustaining their suppressive function, while inhibiting glycolysis in Th17 cells, thereby curbing their pro-inflammatory potential [76].

#### 2.2.2. Immunomodulatory Effects of Bacteriocins

Bacteriocins, a class of ribosomal synthesized antimicrobial peptides produced by *Lactobacilli* species, play crucial roles in microbial competition and host immune modulation [77]. In addition to the canonical function of inhibiting pathogenic bacteria, these peptides can actively influence host immune responses by directly interacting with immune cells and epithelial barriers [78].

Accumulating evidence demonstrates that bacteriocins can activate macrophages and NK cells, augmenting their phagocytic and cytotoxic capacities while stimulating cytokine secretion. For instance, nisin, a bacteriocin produced by *L. lactis*, can potently enhance macrophage-mediated immune defenses [79]. In addition, in vitro studies reveal that nisin treatment increases macrophage phagocytosis of *Staphylococcus aureus* and elevates the cytokine levels in supernatants such as tumor necrosis factor-α and interleukin-1β [80,81].

Mechanistically, bacteriocins exert immunomodulatory effects by engaging recognition receptors on intestinal epithelial cells and triggering intracellular signaling cascades [82], which can induce the expressions of antimicrobial peptides and pro-inflammatory cytokines and activate the NF-κB pathway, resulting in enhancing intestinal epithelial barrier integrity by modulating tight junction protein expressions, and preventing pathogen translocation [83].

#### 2.2.3. Immunomodulatory Effects of Exopolysaccharides

Exopolysaccharides are carbohydrate compounds secreted by *Lactobacilli* during their growth and metabolic processes, typically permeating into the surrounding medium. A portion of these exopolysaccharides adheres to the microbial cell wall, forming capsules, while another fraction dissolves into the medium, creating mucilage (mucopolysaccharides). Exopolysaccharides exhibit excellent biological activity and safety profiles, making them a subject of significant interest in both the food industry and medical fields [51].

*Lactobacilli*-derived exopolysaccharides containing mannose can repair the intestinal barrier, enhance short-chain fatty acid metabolism, upregulate the expressions of anti-inflammatory factors, and alleviate ulcerative colitis [84]. In addition, the exopolysaccharides composed of phosphate groups, sulfate groups, and uronic acids have also shown strong anti-inflammatory effects [85]. The molecular weights of exopolysaccharides produced by *Lactobacilli* are closely associated with their roles in modulating inflammation. High-molecular-weight EPS plays a significant role in alleviating the symptoms of inflammatory diseases, whereas low-molecular-weight EPS often acts as a facilitator in the onset and progression [86].

Therefore, the various metabolites produced by *Lactobacilli* during growth play crucial roles in the immune regulatory process. Based on the current research basis, future research can be carried out in the following directions: 1. The analysis of immune regulatory networks played by metabolites alone or in combination at the cellular and molecular levels. 2. The influence of the growth environment and host factors on the enrichment of *Lactobacilli* metabolites. 3. The research and development of high-efficiency enrichment and stabilization technology of *Lactobacilli* metabolites. 4. The development of personalized functional food combining *Lactobacilli* and metabolites based on the physiological characteristics and health needs of different populations. These findings will comprehensively promote *Lactobacilli* applications in the field of immune regulation and facilitate the innovative development of the food and health industries.

## 3. Factors Influencing Immunomodulatory Activity of Lactobacilli

### 3.1. Lactobacilli Species Differences

Significant variations between and within species are observed in the immunomodulatory capabilities of *Lactobacilli*. Comparative studies on *L. rhamnosus* isolates from diverse ecological niches have revealed strain-specific differences in immune cell activation and cytokine secretion patterns, which can be attributed to genomic diversity and evolutionary adaptations to distinct environmental pressures [87,88]. Genomic analyses can underscore the molecular basis for these variations, demonstrating that different gene expressions among *Lactobacilli* strains can directly influence their immunomodulatory activities [89,90]. Specific strains may harbor unique gene clusters encoding metabolites with specialized immunomodulatory properties, while others diverge in the expression of surface-exposed proteins that mediate interactions with host immune receptors [91]. Moreover, the evolutionary path of a strain and its adaptation to specific environmental niches significantly influence its immunomodulatory capabilities. Strains that have experienced extended selection within certain ecological contexts may develop immunoregulatory strategies that are dependent on environmental or host-specific factors, leading to diverse functional outcomes under different conditions [92,93].

### 3.2. Host Factors

The host’s age significantly influences the immunomodulatory effectiveness of the *Lactobacilli* species. In infancy and early childhood, which are key stages characterized by the development of the immune system, *Lactobacilli* strains are essential in guiding the maturation of immune organs and the functional specialization of immune cells [94]. In elderly populations, where immunosenescence leads to a gradual decrease in immune function, *Lactobacilli*-based interventions might focus on maintaining immune balance and enhancing resistance to pathogens [95]. The physiological conditions of the host, particularly in cases of inflammation-related diseases, significantly impact the immunomodulatory effects of *Lactobacilli*. In LPS-induced inflammation hosts, the richness and activity of *Lactobacilli* are suppressed, which is relevant to the high expressions of inflammatory cytokines including IL-6, TNF-α, and IL-1β [96]. However, it should not be ignored that there is also some *Lactobacilli* enrichment that triggers inflammatory responses, such as native mitral valve infective endocarditis induced by *L. jensenii* [97]. To enhance treatment effectiveness in these scenarios, personalized administration approaches are necessary, taking into account factors like strain choice, dose, and method of delivery [98]. Furthermore, the interplay between the host’s indigenous gut microbiota and *Lactobacilli* strains represents a complex ecological determinant of immunomodulation. The preexisting microbial community can significantly influence *Lactobacilli* colonization dynamics and metabolic activity, thereby shaping its immunomodulatory effects [99].

### 3.3. Environmental Factors

The immunomodulatory activity of *Lactobacilli* was also deeply affected by exposed environmental conditions. The types of bacterial nutrient substrates and growth environmental conditions (culture temperature, pH, etc.) could regulate the growth characteristics of *Lactobacilli* [100], which can dictate bacterial growth kinetics, enzyme activity, and metabolic flux, thereby impacting the synthesis of immunomodulatory components such as peptidoglycan, lipoteichoic acid, and short-chain fatty acids. Optimal temperatures and pH conditions are essential to maintain cellular integrity and metabolic efficiency, ensuring maximal production of bioactive substances [101]. Post-cultivation storage conditions are equally critical for preserving the immunomodulatory potential of *Lactobacilli* preparations. Exposure to adverse storage environments, including elevated temperatures or humidity, can compromise bacterial viability and structural integrity, leading to reduced bioactivity of immunomodulatory molecules [102].

However, the influence mechanisms of *Lactobacilli* immunomodulatory activity still need to be further studied: 1. The molecular influence of the genetic and phenotypic diversity of different *Lactobacilli* strains on the immunomodulatory mechanisms. 2. The relationship between the population dynamics of *Lactobacilli* in the host ecosystem and immunomodulatory function. 3. The influence of environmental factors on the precise molecular pathways of immune regulation in lactic acid bacteria and the interaction of multiple factors. This research will help the applications of *Lactobacilli* immunomodulatory products and promote the development of human health.

## 4. Application Strategies of Lactobacilli

### 4.1. Precision Development of Probiotics

The construction of a comprehensive *Lactobacilli* strain library, underpinned by multi-omics datasets, enables the systematic characterization of genomic features, metabolic potential, and immunomodulatory functionalities through the integrative analysis of metagenomics, transcriptomics, metabolomics, and immunomics data [103], which will facilitate the analysis of strain-specific immunomodulatory phenotypes, including anti-inflammatory, pro-inflammatory, and immune tolerance-inducing capacities. Metagenomic analysis of *L. rhamnosus* has revealed a distinct gene cluster associated with exopolysaccharide biosynthesis, which is strongly correlated with immunoregulatory properties [104]. Functional assays demonstrated that *L. rhamnosus* activates dendritic cells via the TLR2 signaling axis, thereby promoting interleukin-10 secretion and fostering immune tolerance [105]. In addition, in order to identify strains with specific immune-modulating capabilities, in vitro immune cell co-culture systems—including dendritic cells, regulatory T cells, and macrophages—can be utilized to screen the *Lactobacilli* species. Therefore, the organic integration of multiple omics can provide comprehensive data assistance in explaining the molecular mechanisms of *Lactobacilli*-induced immune regulation and help patients select appropriate strains for treatment.

### 4.2. Gene-Editing-Driven Engineering of Lactobacilli

Gene-editing technologies, characterized by their ability to precisely manipulate genomic sequences, offer unprecedented opportunities to enhance the immunomodulatory potential of the *Lactobacilli* species [106]. By deploying site-directed mutagenesis, gene knock-in/out strategies, or pathway engineering, researchers can rationally modify immune-related genetic loci in *Lactobacilli*, thereby redefining host–microbe interactions. Key targets include genes encoding surface-exposed proteins and metabolic pathways that govern immune cell activation, cytokine secretion, and immune tolerance induction. The genetic optimization of genes involved in surface protein expression or metabolite biosynthesis can augment *Lactobacilli*-induced immune signaling. Studies have demonstrated that the targeted manipulation of immunoregulatory pathways in *Lactobacilli* can enhance the capacities to activate dendritic cells and T cell subsets or suppress excessive inflammation [107,108]. This approach holds promise for developing novel *Lactobacilli* strains with maximum therapeutic efficacy while minimizing off-target effects.

### 4.3. Nanocarrier Systems

The utilization of nanocarrier systems to potentiate the targeted encapsulation and protection of *Lactobacilli* metabolites represents a cutting-edge strategy in biomedical engineering, which entails encapsulating bioactive metabolites—such as short-chain fatty acids and exopolysaccharides—within nanomaterials, including chitosan and alginate, to mitigate their degradation by gastric acid and digestive enzymes during transit through the gastrointestinal tract [109]. Chitosan-alginate nanoparticles have been demonstrated to enhance the site-specific delivery efficiency of short-chain fatty acids to the colonic microenvironment, ensuring their bioavailability at the intended therapeutic locus [110]. In addition, these nanocarriers can also perform targeted surface functionalization, such as M-cell targeting peptides [111]. In addition, the nanoencapsulated systems can also be directly applied to probiotics such as *Lactobacilli* to improve their survival ability in complex environments and promote their applications in functional food development. Therefore, the protective encapsulation and targeted delivery not only preserve the biological activity of *Lactobacilli* and the metabolites but also enhance their interaction with immune cells and epithelial barriers, optimizing their immunomodulatory potential.

**Figure 2:**
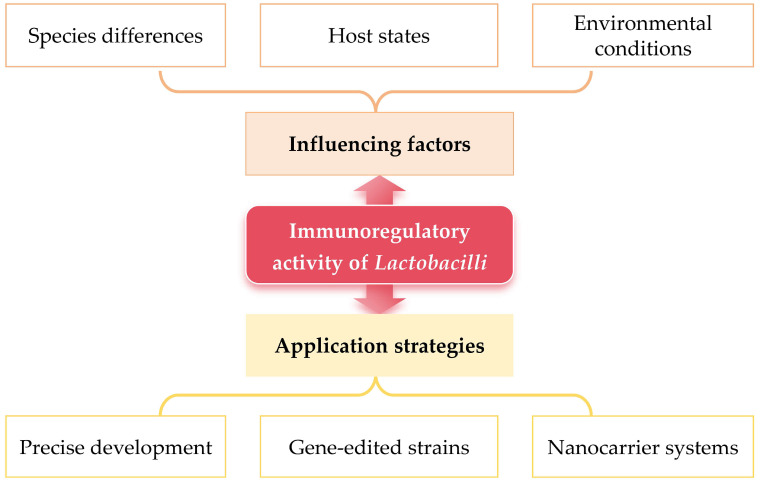
Influencing factors and application strategies of *Lactobacilli* exerting immunomodulatory activity.

Therefore, as shown in Figure 2, in order to fully take advantage of the immunomodulatory efficacy mediated by *Lactobacilli*, in addition to comprehensively considering the key influencing factors, it is also necessary to systematically construct compatible application strategies, forming a complete system in which factors analysis and application strategies complement each other.

## 5. Summary and Prospects

### 5.1. Summary

In summary, *Lactobacilli*, as prominent probiotic bacteria, exhibit remarkable immunomodulatory capacities with the bacterial antigens (including peptidoglycan, lipoteichoic acid, polysaccharides, and surface proteins) and the metabolites (such as short-chain fatty acids, bacteriocins, and exopolysaccharides) as the key immune components. The immunomodulatory effects can be influenced by multiple factors including species-specific differences, host-related factors, and environmental factors. In addition, various strategies are proposed to enhance the immunomodulatory potential, such as probiotics precision development, gene-editing-driven engineering approaches, and nanocarrier systems utilization. This paper will significantly contribute to the development of innovative intervention strategies centered on *Lactobacilli* in the food and medical industries in creating probiotic products with immune-enhancing properties and offer theoretical support for the interdisciplinary advancement of microbiology, immunology, food science, and related fields. However, due to the complexity of the immune system, fully understanding the mechanisms underlying *Lactobacilli*-mediated immune regulation remains challenging. In addition, most of the research in this review was performed in vitro or in animal models, and more clinical trials are still needed to validate the efficacy and safety of *Lactobacilli*-based therapies in humans.

### 5.2. Prospects in the Food Industry

In the food industry, *Lactobacilli* exhibits significant potential for development as functional foods with immune-enhancing properties [112]. Currently, consumer demand for such functional foods is on the rise. The well-characterized *Lactobacilli* strains could be integrated into various food products, including dairy items, fermented beverages, and snacks, which could ensure the palatability of food and satisfy consumer taste preferences, and effectively promote consumer immune health [113,114]. Furthermore, ongoing advancements in innovative food processing technologies would play pivotal roles in maintaining the viability and immunomodulatory activity of *Lactobacilli* throughout production and storage, which would facilitate the expansion of food categories capable of delivering health benefits [110], offering consumers more diverse options and propelling the functional food market toward a healthier and more dynamic trajectory [115].

### 5.3. Prospects in the Pharmaceutical Industry

In the pharmaceutical industry, further research can focus on the potential of developing innovative treatments for immune-related diseases utilizing *Lactobacilli*. By detecting and analyzing the gut microbiota compositions and immune status of individual patients, personalized probiotic formulations could be designed to restore the disrupted immune equilibrium in the gut and mitigate inflammatory responses, thereby promoting tissue repair processes [116]. In managing patients with autoimmune diseases, the negative immunomodulatory mechanism mediated by *Lactobacilli* can effectively suppress excessive immune activation [117]. Moreover, as our comprehension of the gut–brain axis continues to expand, *Lactobacilli* is anticipated to serve as a potent tool for modulating the immune system and positively addressing mental health issues associated with immune dysregulation [118].

## Data Availability

No new data were created or analyzed in this study. Data sharing is not applicable to this article.
